# Sequentially coupled musculoskeletal multibody and finite element simulation for biomechanical modeling of the human masticatory system

**DOI:** 10.3389/fbioe.2026.1809299

**Published:** 2026-06-23

**Authors:** Iman Soodmand, Ann-Kristin Becker, Jan-Oliver Sass, Christopher Jabs, Michael Dau, Rainer Bader, Maeruan Kebbach

**Affiliations:** 1 Research Laboratory for Biomechanics and Implant Technology, Department of Orthopedics, Rostock University Medical Center, Rostock, Germany; 2 Department of Oral, Maxillofacial Plastic Surgery, Rostock University Medical Center, Rostock, Germany

**Keywords:** boundary conditions, finite element analysis, mandibular biomechanics, material properties, muscle attachment area, musculoskeletal multibody simulation, subject-specific modelling

## Abstract

**Introduction:**

Finite element (FE) models of the mandible are widely used for biomechanical analysis and preoperative planning in craniomaxillofacial surgery. However, their predictive reliability depends on a realistic representation of geometry, material properties, boundary conditions, and muscular loading, for which no clear consensus currently exists. Therefore, we developed a sequentially coupled computational framework that integrates boundary conditions from a validated inverse-dynamic, scaled-generic musculoskeletal multibody simulation (MMBS) of the human masticatory system under unilateral clenching into a subject-specific, quasi-static FE model of the mandible.

**Methods:**

The MMBS model was scaled to a mandibular geometry reconstructed from computed tomography (CT) images. Time-dependent muscle forces and mandibular landmark displacements were transformed from the MMBS to the FE coordinate system and implemented as loading and boundary conditions, respectively. Heterogeneous bone material properties were assigned from CT data. Additionally, a baseline FE model, using common literature assumptions, including static generic muscle forces, fixed boundary conditions, and homogeneous bone material properties, was defined. Starting from this baseline model, sensitivity analyses of the calculated strain and stress distributions in clinically relevant regions of interest were conducted to quantify the influence of bone material properties, applied muscle forces, and the muscle attachment area, thereby isolating the effect of each modeling advancement and demonstrating the value of the proposed MMBS-FE framework.

**Results:**

The sequentially coupled MMBS-FE framework predicted realistic mandibular biomechanics with the highest von Mises stresses and logarithmic strains occurring in the balancing-side condyle and the working-side molar region. Bone material properties were the dominant source of variation, causing changes of peak stress up to 161% and peak strain up to 723%, followed by more modest effects from applied muscle forces (up to 31%) and a negligible influence of muscle attachment area definition.

**Discussion:**

These findings highlight the critical importance of realistic modeling of heterogeneous bone material properties and personalized, time-dependent coordinated muscle forces that serve as loading in subject-specific mandibular FE models. This proposed MMBS-FE model serves as a proof-of-concept framework and offers recommendations for biomechanical modeling of the human masticatory system under unilateral clenching conditions. By incorporating these recommendations, researchers can improve pre-clinical evaluation of craniomaxillofacial implants and surgical strategies.

## 1 Introduction

Over the past decades, surgical interventions involving the mandible bone have evolved substantially, and computational modeling has increasingly been used to support biomechanical analysis, pre-clinical evaluation, and, in selected cases, preoperative planning (Lisiak-Myszke et al., 2020; Aftabi et al., 2023; Falcinelli et al., 2023). Major developments include improved techniques for mandibular reconstruction (Kargarnejad et al., 2020; Ruf et al., 2022), osteotomy procedure (Chang et al., 2020; Luo et al., 2023), management of maxillofacial trauma (Orassi et al., 2021; Maintz et al., 2022; Gupta et al., 2023), temporomandibular joint (TMJ) replacements (Ackland et al., 2018; Pinheiro et al., 2021; Woodford et al., 2024), and the design and positioning of dental implants and restorations (Azcarate-Velázquez et al., 2019; Lin and Su, 2020). These interventions aim to restore both function and aesthetics in patients affected by craniofacial deformities, or dental pathologies (Lisiak-Myszke et al., 2020; Falcinelli et al., 2023; Yang et al., 2024). Despite these advances, many patients still experience postoperative complications, including failure of medical devices or limited functional recovery (Radi et al., 2018; Block and Christensen, 2021). This highlights the existing challenges in the surgical planning and the need for improved implant designs, material selection, and personalized preoperative strategies (Ramos et al., 2014; Dhatrak et al., 2019; Saini et al., 2020; Merema et al., 2021; Sass et al., 2024b; Soodmand and Becker et al., 2024). In this regard, a pre-clinical evaluation under more realistic conditions is an important step, enabling optimization of device performance and adaptation to biomechanical requirements (Chang et al., 2018; Merema et al., 2021).

Conventional experimental approaches typically apply static and uniaxial loading and are constrained by limited donor availability (Hannam et al., 2008; Ben Achour et al., 2024; Zhong et al., 2025). Compared to this, computational approaches provide a more realistic representation of the dynamic loading and boundary conditions experienced by the masticatory system during daily activities (Zee et al., 2007; Saini et al., 2020). In addition, by integrating subject- or cohort-specific characteristics such as bone morphology (Sass et al., 2025) and material properties (Dhatrak et al., 2019; Sass et al., 2025), computational models enable higher fidelity, personalized analyses that better reflect realistic behavior. The limited use of such realistic and personalized assessments in current pre-clinical evaluations in the craniomaxillofacial field may partly explain the persistent incidence of implant failures and incomplete functional restoration after treatment (Chang et al., 2018; Merema et al., 2021).

In this context, musculoskeletal multibody simulations (MMBS) and finite element (FE) analysis have been widely employed to computationally investigate the biomechanics of the human masticatory system and its interaction with medical devices (Ackland et al., 2015; Sagl et al., 2019; Woodford et al., 2024; Andres et al., 2025; Sass et al., 2025). On one hand, MMBS operates at the organ-level, efficiently estimating muscle forces, joint kinematics, and reaction forces of the mandible and providing realistic boundary conditions during active movements (Zee et al., 2007; Skipper Andersen et al., 2017; Sagl et al., 2019). On the other hand, FE analysis focuses on the tissue-level mechanics, offering detailed information on localized stress or strain distribution within bone volume and medical devices, as well as their bidirectional mechanical interaction (Lisiak-Myszke et al., 2020; Aftabi et al., 2023; Falcinelli et al., 2023). A sequential coupling of MMBS and FE analysis, therefore, enables an integrated approach that combines the capabilities of both domains, and may improve the physiological relevance of the biomechanical simulations of masticatory system (Dao, 2017; Nispel et al., 2023; Sass et al., 2024a; Dastgerdi et al., 2025).

Despite progressive advancements of such computational approaches for the human masticatory system over recent decades, there is still no consensus on key model input parameters (Merema et al., 2021). In most FE models, bone material properties are simplified by assuming homogeneous behavior, despite strong dependence on the specific subject and anatomical site (Helgason et al., 2008a; Xin et al., 2013; Knowles et al., 2019; Hussein and Alruthea, 2021; Soodmand and Becker et al., 2024). Celik et al. reviewed the use of homogeneous material properties and called for further studies to confirm the accuracy of FE studies in dental research (Celik et al., 2022). Soodmand and Becker et al. provided a detailed comparison of heterogeneous material models for the mandible, identified 12 distinct heterogeneous material definitions, reported a broad range of Young’s moduli for the bone, and discussed their biomechanical implications (Soodmand and Becker et al., 2024). They also highlighted the need for comparative FE studies to assess the effect of material model choice on the mechanical response of the bone, calculated by FE models. Moreover, Hussein and Alruthea compared homogeneous and heterogeneous material assignments based on three different material models for estimating subject-specific mandibular properties and showed that different empirical relationships can lead to markedly different stress and strain predictions, thereby raising concerns about the reliability of such simulations, and the risk of misleading clinical conclusions (Hussein and Alruthea, 2021). Collectively, these studies highlight a lack of consensus regarding the implementation of heterogeneous bone material modeling. In addition, several studies have employed simplified boundary conditions, such as fully fixed condyles or fixed teeth at the clenching position (Lin and Su, 2020; Orassi et al., 2021) and in some studies, analyses were limited to a partial mandible segment rather than the entire bone (Korabi et al., 2017; Didier et al., 2020) which does not replicate a real condition and may lead to inaccurate findings. Some researchers have demonstrated that reliable FE predictions require accurate representation of muscle force magnitudes, directions, and attachment areas across the full mandible (Breuer et al., 2020; Saini et al., 2020; Orassi et al., 2021; Andres et al., 2025). In this context, Nelson proposed a set of masticatory muscle forces, the corresponding insertion coordinates, and the activation scaling factors for unilateral clenching (Nelson, 1986). Korioth and Hannam added the fiber directions, or the so-called direction cosines, calculated from 3D muscle attachment coordinates (Korioth and Hannam, 1994). These muscle vectors have been extensively used as loading conditions in subsequent FE studies (Lin and Su, 2020; Orassi et al., 2021; Pinheiro et al., 2021; Ruf et al., 2022; Luo et al., 2023; Yang et al., 2024). However, these forces were derived from a single healthy subject, necessitating personalization when applied to models of other individuals, especially with known pathologies (Korioth and Hannam, 1994; Orassi et al., 2021). To achieve greater personalization, Zheng et al. incorporated the physiological cross-sectional area as a key determinant of masticatory muscle functionality (Zheng et al., 2019).

Previously, Moazen et al. coupled MMBS and FE analysis in a lizard skull, transferring time-varying muscle, bite, and joint loads across a biting cycle and demonstrating the value of integrated loading in an animal model (Moazen et al., 2008). Dao later proposed a multi-scale mandible workflow linking MMBS, FE analysis, and agent-based remodeling, although the mandible application remained generic and focused on bone metabolic processes during jaw opening (Dao, 2017). In a patient-specific clinical application, Ackland et al. employed the same approach to evaluate a custom TMJ prosthesis and its geometric design sensitivity during chewing and biting, with a primary focus on implant performance (Ackland et al., 2018). Furthermore, Sagl et al. developed a MMBS of the human masticatory system that integrates rigid-body mandible mechanics with an FE representation of the TMJ disc and elastic cartilage in a single framework (Sagl et al., 2019). Most recently, Woodford et al. introduced an integrated experimental-computational framework that combines CT-registered optoelectronic motion tracking with MMBS to estimate subject-specific mandibular joint kinematics, muscle and joint forces under static equilibrium conditions (Woodford et al., 2024). Despite these efforts toward integrated mandibular modeling, no systematic study has yet quantified how uncertainty in model input parameters, e.g., material model or boundary conditions, propagates to specific biomechanical output predictions such as stress and strain of FE simulations of the mandible. Most studies are limited to either animal models, bone remodeling, TMJ soft-tissue mechanics, implant-specific applications, or static-equilibrium loading, and therefore do not provide a sequentially coupled framework for the native human mandible under more realistic loading and boundary conditions.

Therefore, the first objective of the present study is to develop a computational framework and proof-of-concept that sequentially couples a scaled-generic validated inverse-dynamics MMBS of the human masticatory system with a subject-specific FE model of the mandible to analyze a representative daily activity of unilateral clenching scenario. The second objective is to evaluate the effect of current uncertainty in key input parameters of the computational model of the masticatory system by conducting a sensitivity analysis on those model inputs and to quantify their influence on simulation outcomes. The findings of this study are expected to guide researchers in developing more realistic and reproducible computational models of the masticatory system by providing evidence-based discussion on the selection of input parameters (Merema et al., 2021). Furthermore, the proposed computational framework offers potential benefits for medical device manufacturers, enabling the optimization of the design and performance of products used in the craniomaxillofacial field (Andres et al., 2025).

## 2 Materials and methods

**FIGURE 1:**
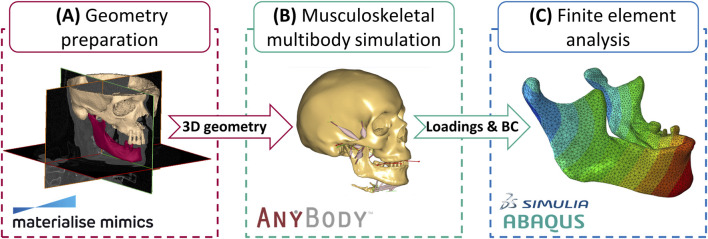
Sequentially-coupled simulation framework of the human masticatory system. For the proposed methodology, first, the **(A)** geometry preparation and segmentation of the mandible bone is performed based on CT medical images. This is followed by **(B)** a scaled-generic musculoskeletal multibody simulation for unilateral left side clenching from which muscle forces and displacements can be exported. Those serve as loadings and boundary conditions (BC) for the **(C)** finite element analysis of the mandible.

A three-dimensional computational model of a human masticatory system was developed using 3D reconstructed models of the bones. Musculoskeletal multibody simulation (MMBS) and finite element (FE) analysis were sequentially coupled to form a simulation framework (Figure 1). This framework was employed to estimate muscle forces and kinematics as well as to investigate localized mechanical responses of the mandible bone, including strain and stress distribution during the unilateral clenching on the left first molar tooth.

### 2.1 Imaging and data acquisition

The basis geometry for this study was obtained from a set of existing CT images (Revolution CT, GE HealthCare) from a 39-year-old male subject. Ethical approval for this study was obtained from the local ethics committee at Rostock University Medical Center (Approval ID: A 2023-0042). The medical images include the mandible, hyoid, and part of the skull; the subject was missing the first molar on both sides but had no history of temporomandibular joint disorders. A pixel spacing of 0.56 × 0.56 mm and a slice thickness of 0.625 mm were used for the scan, which resulted in 314 CT slices. The CT scan device was calibrated using a phantom (QRM, Moehrendorf, Germany), enabling us to relate the Hounsfield units (HU) of the bone to its apparent density, which is subsequently correlated with Young’s modulus to consider the heterogeneous bone material properties (Soodmand and Becker et al., 2024).

### 2.2 Reconstruction and segmentation

The CT images were used for segmentation and reconstruction of 3D surface models in Mimics 25.0 software (Materialise, Leuven, Belgium) and exported as stereolithography (STL) files (Kluess et al., 2009). Each bone was segmented and reconstructed into a unified 3D model, with separate manual labeling of cortical and trabecular bone tissues. Post-processing of these 3D surface models was performed in Geomagic Studio v.2013 (Geomagic Inc., Morrisville, NC, United States), where the quality of the bone geometries was further refined. The processed 3D geometries were then imported into the AnyBody modeling system™ 7.4 (AnyBody Technology A/S, Aalborg, Denmark) as the target geometry for MMBS and Abaqus/CAE v.2022 (Dassault Systèmes Simulia Crop, Vélizy-Villacoublay, France) for FE analysis, where additional model parameters were defined for the simulations as follows.

### 2.3 Musculoskeletal multibody simulation of the unilateral clenching

**FIGURE 2:**
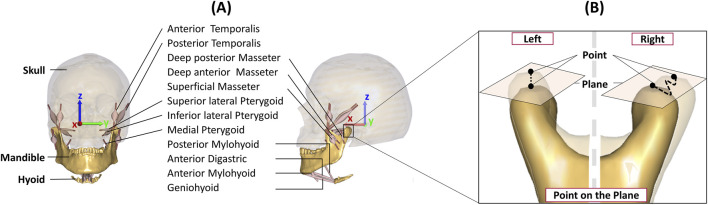
Illustration of the scaled-generic point-on-the-plane musculoskeletal rigid body model of the human masticatory system in AnyBody Modeling System^TM^. **(A)** The model includes the skull (transparent), mandible, and hyoid bones, along with 24 Hill-type muscle elements representing the main and accessory muscles of mastication. The mandible bone was scaled from the generic model to match the target mandible of the studied subject in the present study, reconstructed from CT images. Views are shown in the frontal plane and the sagittal plane. The x-direction points anteriorly (red arrow), the y-direction laterally (green arrow), and the z-direction superiorly (blue arrow). Dark and light red volumes represent the muscle bundles, and the yellow bodies represent the bones. **(B)** The point-on-the-plane constraint is illustrated, highlighting that the left condyle is fixed in mediolateral direction, while the right condyle is free to move along the plane. The plane is inclined 30° downward relative to the occlusal plane and tilted 5° medially (Langenbach and Hannam, 1999; Zee et al., 2007).

A validated MMBS of the human masticatory system from Anybody Modeling System™ (Zee et al., 2007) was used in the present study (Figure 2). The 3D mandible bone geometry in the model was scaled and morphed to match the anthropometric data of the target subject. An inverse dynamics analysis was performed to estimate muscle forces and mandibular landmark displacements during a unilateral left-side clenching scenario. These data served as boundary and loading conditions for the subsequent FE analysis.

#### 2.3.1 Model topology

This model contains three rigid bodies: the skull, the mandible, and the hyoid bones (Figure 2). The coordinate system, as shown in Figure 2, was defined with the x-direction pointing anteriorly, the y-direction pointing laterally, and the z-direction pointing superiorly and was close to the parasagittal plane located at the cranial base between the left and right condyles, rigidly fixed to the skull. The skull and the hyoid bones were fixed with zero degrees-of-freedom (DOF) and had no movements throughout the simulations. The mandible bone articulates with the skull through point-on-plane constraints (Zee et al., 2007). More specifically, the condyles were constrained to move on a plane, inclined 30° downward relative to the occlusal plane and tilted 5° medially (Langenbach and Hannam, 1999; Zee et al., 2007). Therefore, the model is also known as the point-on-the-plane model (Zee et al., 2007) (Figure 2B). In addition, the left condyle was restrained from movement in medial-lateral direction. This resulted in a mandible with three DOFs to provide mouth opening, retraction, protraction and rotation; for example, to simulate the chewing motion. Captured motion data from the repository model in the AnyBody modeling system™ for a unilateral clenching scenario drive these three DOFs (Zee et al., 2007), while a corresponding measured time-dependent occlusal bite force with a maximum of 441 N, derived from the repository model in the AnyBody modeling system™, was applied to the position of left first molar. This force magnitude matches the range for maximum bite force presented in the meta-analysis by Daqiq et al. for a healthy dentulous population aged 20–60 years old (Daqiq et al., 2025). According to this model definition, the left side of the mandible is considered the working side and the right side the balancing side throughout the present study.

#### 2.3.2 Model scaling and calibration

**FIGURE 3:**
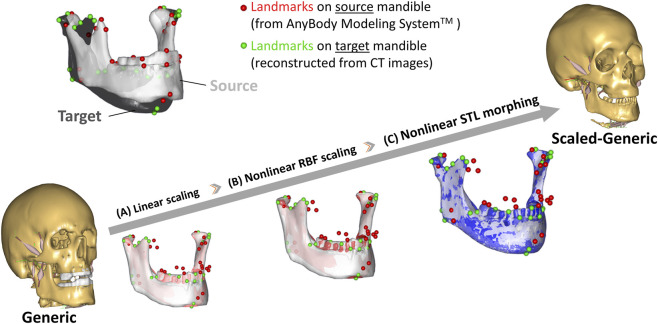
Illustration of the CT-based scaling and morphing process of the generic musculoskeletal multibody mandible model of the AnyBody Modeling System^^™^^ to the target mandible from CT-based reconstruction. **(A)** Linear scaling based on user-defined anatomical landmarks on both source and target mandibles, **(B)** nonlinear radial basis function (RBF) scaling based on anatomical landmarks on both source and target mandibles (AnyBody Technology, 2025b), **(C)** and stereolithography (STL) based morphing of the bone surface (AnyBody Technology, 2025b).

The mandible, skull, and muscle paths were scaled anisotropically to ensure accurate anatomical representation. For this, the bone geometry of the generic mandible model of the AnyBody Modeling System™ was scaled to the target bone geometry of the present study. This was done using a 3-step scaling approach, shown in Figure 3: (A) linear scaling based on user-defined anatomical landmarks, (B) nonlinear radial basis function (RBF) scaling based on anatomical landmarks, and (C) STL morphing of the bone surface to align the 3D reconstructed geometry of the target mandible bone geometry (Marra et al., 2015; Dejtiar et al., 2020; AnyBody Technology, 2025b). The scaling landmarks were selected from established cephalometric and morphometric mandibular landmarks introduced in the literature (Williams and Richtsmeier, 2003; Franklin et al., 2006; Bejdová et al., 2013). A subset of these landmarks was used in the three-step scaling and morphing procedure. The subject-specific reconstructed skull and hyoid bones, derived from CT images, were integrated into the model to refine the muscle attachment sites, lengths, lines of action, and temporomandibular joint centers. This was done to achieve personalized and more accurate musculoskeletal modeling (She et al., 2018). The correctness of adjusted muscle attachment sites and lines of action was proved with anatomical definitions and further evaluated visually by an experienced surgeon. Following the scaling and personalization, the muscle-tendon units were calibrated. For that, the length of serial elastic element of the Hill-type muscles was adjusted at an interincisal separation of 12 mm (Langenbach and Hannam, 1999). This adjustment aimed to ensure maximal muscle force generation, assuming the subject exerted maximum clenching force. It was also assumed that, at initial condition, the passive element of the muscle contributed minimally, as no external load was yet applied to the mandible (Manns et al., 1979; AnyBody Technology, 2025a).

#### 2.3.3 Inverse dynamics and muscle force calculation

The model includes 24 musculotendon actuator elements (Figure 2A) representing the main muscles of mastication including Temporalis [anterior (AT) and posterior (PT)], Masseter [deep posterior (DPM), deep anterior (DAM), and superficial (SM)], Pterygoid [medial (MP), inferior lateral (ILP), superior lateral (SLP)] and the accessory muscles of mastication including Mylohyoid (anterior and posterior), anterior Digastric and Geniohyoid. Each actuator was modelled as a Hill-type muscle consisting of contractile, parallel elastic and serial elastic elements (Heinen et al., 2016). For all actuators, the peak isometric force (*F*_max_) and optimal fiber length were selected based on previous experiments on human specimens (Koolstra and van Eijden, 2004; Zee et al., 2007). The line of action for each muscle actuator was defined as the vector connecting the origin and insertion of the centroid of the musculotendon element. Given the prescribed time-dependent mandible kinematics, together with the anthropometric and mandibular geometry of the studied subject, inverse-dynamics solves the equations of motion to compute the joint moments and reaction forces at the TMJ. The bite force was then decomposed in AnyBody Modeling System™ into muscle and joint forces using an static optimization problem that minimized a third order polynominal objective function, $G\left(f^{\left(M\right)}\right)$ , Equations 1–3, subjected to force ( f ) and moment ( M ) equilibrium constraints. This optimization problem solved the muscle redundancy problem, recruited the masticatory muscles to perform the desired unilateral clenching task, and allowed symmetric muscle function (Rasmussen et al., 2001).

$$\min_{f}G\left(f^{\left(M\right)}\right),G\left(f^{\left(M\right)}\right)=\sum_{i=1}^{n^{\left(M\right)}}{\left(\frac{f_{i}^{\left(M\right)}\left(t\right)}{f_{\max,i}^{\left(M\right)}}\right)}^{3}$$ (1)

subject to

Cf=M (2)

$$\text{and }f_{i}^{\left(M\right)}\geq 0,i=1,\ldots ,n^{\left(M\right)}$$ (3)

where $f_{i}^{\left(M\right)}\left(t\right)$ is the time-dependent muscle force, C is the matrix of coefficients depending on the current position of the body segments, $n^{\left(M\right)}$ equals 24, the number of muscles considered in the MMBS model, and *i* serves as a counter.

The scaled-generic, calibrated model was used for the simulation, which was carried out on an off-the-shelf desktop computer with a single processor (3.3 GHz, Core i5, 32 GB RAM), and the wall-clock time was 14 s.

After solving the MMBS, the resultant muscle forces and the displacement of three selected mandibular landmarks, including two condyles and the clenching position, were exported as time-vectors in the global coordinate system of the MMBS model (see Supplementary Material Table SA1; Supplementary Figure SA1). After transforming these data from the MMBS coordinate system to the CT scan coordinate system, they served as personalized loading and boundary conditions to generate the sequentially coupled FE model.

### 2.4 Finite element simulation

**FIGURE 4:**
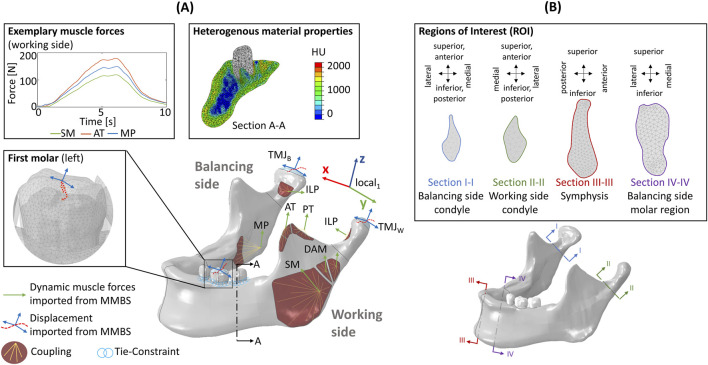
**(A)** Proposed sequentially coupled MMBS-FE modeling framework of the human masticatory system for unilateral clenching. Local coordinate system origin and direction imported from musculoskeletal multibody simulation (MMBS), (local_1_). The right side is considered as balancing, and the left side as the working side. Muscle forces are applied *via* reference points coupled to the muscle attachment areas. Green arrows indicate forces and direction imported from the multibody simulation (SM, Superficial Masseter, DM, Deep Masseter, AT, Anterior Temporalis, PT, Posterior Temporalis, ILP, Inferior lateral Pterygoid, MP, Medial Pterygoid). Three exemplary muscle forces are shown in the graph (SM, AT, MP). Blue coordinate systems indicate displacements on three anatomical points (TMJ center (balancing and working side), First Molar (working side)) imported from multibody simulation. A detailed view of the left first molar shows the imported displacement. Material properties are assumed to be heterogeneous based on the data from medical images (Keyak et al., 1998) considering the Hounsfield units (HU) as shown in the detailed view. Regions with high Young’s modulus are represented in red and light green, whereas low Young’s modulus areas appear in dark blue. Teeth are assumed to be fully osseointegrated and therefore tie-constrained. **(B)** Region of interest investigated for the human mandible. The balancing and working sides condyles, symphysis and balancing-side molar region are shown in cut views.

A muscle-driven, quasi-static FE model of the masticatory system during the clenching scenario was created using the FE software Abaqus/CAE Standard v.2022 with the static implicit solver (Figure 4A). The analysis was performed under quasi-static conditions, assuming negligible inertial effects, and was muscle-driven, meaning that muscle forces were applied from the previously described MMBS model as loading conditions. Furthermore, the movement of the mandible bone during clenching was reproduced by importing the kinematics of the three anatomical landmarks from the MMBS as boundary conditions. The FE model aims to simulate the biomechanical responses of the mandible, e.g., logarithmic strain and von Mises stress. The reconstructed 3D geometry of the mandible, the left first molar tooth and its neighboring teeth, including second premolar and second molar, were used to create the FE model (Figure 4A). As the selected subject missed the first left molar tooth, a geometry from a comparable subject was instead included and positioned under the supervision of an experienced surgeon. The teeth were assumed to be fully osseointegrated; therefore, a completely constrained interface was defined between the tooth and the bone, using a tie constraint (Falcinelli et al., 2023).

In addition to the global coordinate system derived from the CT scan, a local coordinate system (local_1_) was defined in the FE model, aligning with the global coordinate system of the AnyBody Modeling System™ (Figure 4). This was necessary to correctly transfer loading and boundary conditions from the MMBS to the FE model.

#### 2.4.1 Regions of interest

Four cross-sections along the mandible were defined as regions of interest (ROIs). Each region was created by partitioning the mandible in the FE software, and the corresponding cross-sectional surfaces are illustrated in Figure 4B. The ROIs were selected based on their clinical relevance and previously reported fracture risk patterns (Liokatis et al., 2021; Gupta et al., 2023; Li et al., 2024). Both condyles (Sections I-I and II-II) were included as ROI because they exhibit high fracture risk due to stress concentrations during masticatory loading (Liokatis et al., 2021; Gupta et al., 2023; Li et al., 2024) and represent common sites for TMJ implant positioning (Ackland et al., 2018; Pinheiro et al., 2021). In contrast, the symphysis (Section III-III) was selected as a low-risk region for fractures (Orassi et al., 2021; Li et al., 2024). The balancing side molar region (Section IV-IV) was added because it corresponds to the balancing side during unilateral clenching and therefore provides a comparative site for stress transmission and dissipation across the mandible (Orassi et al., 2021). Both the symphysis and molar regions are also clinically relevant sites for mandibular reconstruction, particularly following tumor resection (Cheng et al., 2019; Orassi et al., 2021). The selected ROIs were used for the sensitivity analysis and the mesh convergence study.

#### 2.4.2 Meshing and convergence

Meshing was performed in Abaqus/CAE and quadratic tetrahedral elements (C3D10) were used for all geometries. To ensure mesh accuracy, a convergence analysis was conducted based on the maximum von Mises stress in the ROIs. We increased the number of elements progressively by a factor of approximately 1:1.3 to generate finer meshes (Oefner et al., 2021) to examine the sensitivity of the results to six different mandibular meshes, varying from 8 mm to 1.5 mm mean element edge length. Details of generated meshes are listed in Supplementary Material Table SA2 in the Supplementary Material. The mesh convergence study aimed to evaluate the element size to achieve a relative difference for maximum strain and stress of less than 5% between consecutive mesh refinements in the ROIs (see Supplementary Figure SA1) (Oefner et al., 2021). The interacting tooth surfaces were meshed separately with a finer element edge length of approximately 0.6 mm to accurately capture surface details and curvatures. A mesh convergence study was performed with homogeneous material properties (Celik et al., 2022) and with simplified loading conditions (Nelson, 1986), defined as the baseline model, which is described in detail in the following.

#### 2.4.3 Material properties

All materials were considered isotropic and linear elastic. Teeth are considered homogeneous with a Young’s modulus of E_dentin_ = 18.6 GPa and a Poisson’s ratio of ν_dentin_ = 0.31 (Celik et al., 2022). To accurately capture the complex biomechanical behavior of bone tissue, the present study incorporated subject-specific heterogeneous bone properties derived from CT images (Soodmand and Becker et al., 2024). Therefore, the HU values obtained from the corresponding CT images were used to assign spatially varying material properties to the nodes of the FE mesh. The HU values were first mapped to the FE model as virtual nodal temperature values using a custom-written MATLAB script, corresponding to the HU values in the CT scan. In this manner, bone heterogeneity was introduced by defining a virtual, temperature-dependent elastic modulus (Helgason et al., 2008b; Mauck et al., 2016). To reduce inaccuracies caused by the partial volume effect, particularly the artificially low stiffness of surface elements, the highest HU value in proximity to each surface node was used instead (Helgason et al., 2008b). The ash density of the bone was calibrated based on HU values using a linear regression equation (Equation 4) derived from a phantom-based function, establishing a relationship between HUs and ash density (ρ_ash_):

$$\rho_{ash\;}=0.000649\;HU-0.030839$$ (4)

The Young’s modulus (E) was subsequently calculated for each node using one of the most widely accepted material models (Soodmand and Becker et al., 2024) for the mandible, proposed by (Keyak et al., 1998), which accounts for trabecular bone, transition zone, and cortical bone. The Poisson ratio of the cortical bone, trabecular bone, and teeth was assumed to be 0.3 (Celik et al., 2022). A summary of the assigned material properties and the corresponding power law equations is provided in Table 1.

**TABLE 1:** Overview of the isotropic, linear-elastic material properties used in the finite element model of the proposed simulation framework.

Material		Young’s modulus [MPa]	Poisson’s ratio	References
Teeth^hom^	Dentin	18,600	0.31	Celik et al. (2022)
Bone^het^	Cortical	3,653 ρ_app_^2.01^ (ρ_app_ ≥ 1.0)	0.30	Keyak et al. (1998)
	Transition	3,184 ρ_app_ + 469 (0.45 < ρ_app_ < 1.0)		
	Trabecular	11,019 ρ_app_^2.2^ (ρ_app_ ≤ 0.45)		

hom, homogenous; het, heterogeneous.

ρ_app::_ apparent density [g/cm^3^], Keyak et al., 1998 originally reported in ρ_ash_: ash density, transformation made with ρ_ash_ = 0.6 ρ_app_ (Schileo et al., 2008).

#### 2.4.4 Boundary conditions

The mandible was driven by the combined action of the masticatory muscles and the kinematics of the three anatomical landmarks exported from the MMBS (see Section 2.3). In this regard, muscle forces, estimated by the MMBS, were applied to the bone in the FE model as amplitude-modulated concentrated loads to build a quasi-static FE model. Each muscle force was applied at a reference point, which was coupled to the corresponding muscle attachment area (MAA) on the outer surface of the mandible (Saini et al., 2020; Orassi et al., 2021). Mandibular motion was prescribed through the displacement of points located at the center of the superior articulating surfaces of the condyles and the bite force application point on the left first molar (Figure 4A). The continuum coupling of both the muscle forces and the kinematics to the mandible outer surface ensured a realistic transmission of force and displacement over the anatomical insertion area and minimized artificial stress concentrations (van Essen et al., 2005).

#### 2.4.5 Sensitivity study on finite element modeling parameters

**FIGURE 5:**
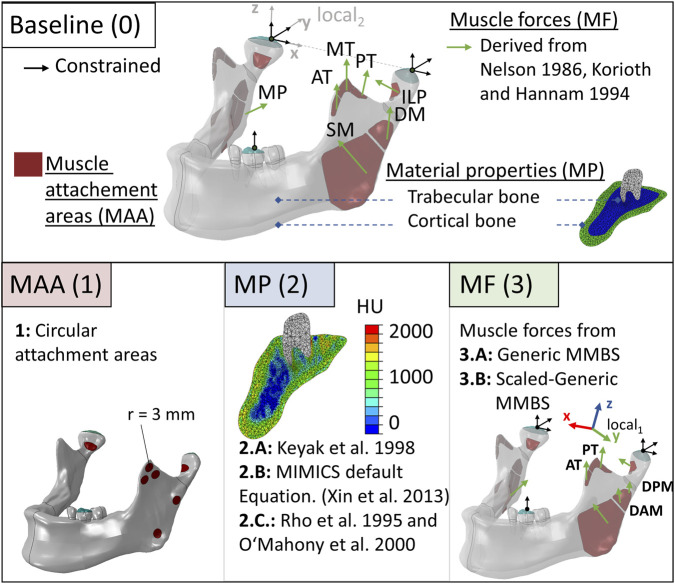
Case studies for the sensitivity analysis on the effect of input parameters to the FE model on the simulation results. Baseline (0) with muscle attachment areas based on frequently quoted literature (Saini et al., 2020), homogeneous material properties for trabecular and cortical bone (Celik et al., 2022) and muscle forces proposed by (Nelson, 1986; Korioth and Hannam, 1994) in the corresponding coordinate system local_2_, as mostly used in literature. Sensitivity analysis includes changing the (1) Muscle attachment areas (MAA) to circular attachments, (2) changing the material properties (MP) to heterogeneous linear-elastic material based on the Hounsfield units (HU) values received from the CT image and (3) the variation of muscle forces (MF) to forces derived from musculoskeletal multibody simulation (MMBS).

To develop the proposed simulation framework in this study, we conducted several sensitivity analyses on the FE model input parameters (Table 2). In this regard, to establish a reference model for comparison, a “Baseline model” was created using the most commonly adopted modeling parameters reported in previous FE studies of the mandible in the literature (Saini et al., 2020; Merema et al., 2021; Orassi et al., 2021; Falcinelli et al., 2023). Following the development of this Baseline model (Case 0), three case studies were defined for sensitivity analysis, focusing on (Case 1) muscle attachment areas, (Case 2) bone material properties and (Case 3) personalized muscle forces. A schematic summary of all these case studies, including Baseline and varied input parameters in cases 1–3, is provided in Figure 5 and Table 2. For each case, only a single input parameter was varied to study its isolated effect on the results.

**TABLE 2:** Studied parameters in the sensitivity analysis.

Case number	Muscle attachment areas	Material properties Youngs modulus [MPa]			Muscle forces	
Baseline Case 0	Anatomically defined (Saini et al., 2020)	​	Bone^hom^ Celik et al. (2022)	E_cort_ = 13,000 E_trab_ = 1,370	Literature-based (Nelson, 1986; Korioth and Hannam, 1994)	
Case 1	**Circles with 3 mm radius** Sass et al. (2025)	​	Bone^hom^ Celik et al. (2022)	E_cort_ = 13,000 E_trab_ = 1,370	Literature-based (Nelson, 1986; Korioth and Hannam, 1994)	
Case 2	Anatomically defined (Saini et al., 2020)	**A**	**Bone** ^**het**^ Keyak et al. (1998)	**E**_**cort**_ = **3,653 ρ** ^**2.01**^ (ρ ≥ 1.0)	Literature-based (Nelson, 1986; Korioth and Hannam, 1994)	
				**E**_**trans**_ = **3,184 ρ + 469** (0.45 < ρ < 1.0)		
				**E**_**trab**_ = **11,019 ρ** ^**2.2**^ (ρ ≤ 0.45)		
		**B**	**Bone** ^**het**^ Xin et al. (2013)	**E** = **−388.8 + 592.5 ρ**		
		**C**	**Bone** ^**het**^ Rho et al. (1995), O'Mahony et al. (2000)	**E**_**cort**_ = **−23,930 + 24,000ρ** (ρ > 1.0)		
				**E**_**trab**_ = **2,349 ρ** ^**2.15**^ (ρ ≤ 1.0)		
Case 3	Anatomically defined (Saini et al., 2020)	​	Bone^hom^ Celik et al. (2022)	E_cort_ = 13,000 E_trab_ = 1,370	**A**	**Imported from generic MMBS**
					**B**	**Imported from scaled-generic MMBS**

hom, homogeneous; het, heterogeneous; MMBS, Musculoskeletal multibody simulation.

ρ is ρ_app_ [g/cm3], with ρ_ash_ = 0.6 ρ_app_ (Schileo et al., 2008): ρ_ash_ = ash density, ρ_app_ = apparent density.

For all bone material properties: ν = 0.3. Text highlighted with boldface indicates the varied input parameters in each sensitivity case. In Case 1 one variation of muscle attachment areas, in Case 2 three different material properties (A, B, C) and in Case 3 two different muscle forces were studied (A, B).

##### 2.4.5.1 Baseline model (0)

For the Baseline model, linear elastic, isotropic and homogenous material properties were assigned to all components, including the bone, using the most frequently used parameters in the literature E_trab_ = 1,370 MPa, and ν_trab_ = 0.3 for the trabecular bone, E_cort_ = 13,000 MPa, and ν_cort_ = 0.3 for the cortical bone, and E_teeth_ = 18,600 MPa, and ν_teeth_ = 0.31 for the teeth (Celik et al., 2022).

Instead of the muscle forces calculated for the subject of our study using the described scaled-generic MMBS, in the Baseline model, the muscle forces proposed by Nelson for a left unilateral clenching of 385 N on a first molar were applied to the mandible (Nelson, 1986; Korioth and Hannam, 1994). This is the most common approach in the literature over the past decade for modeling loading conditions (Lin and Su, 2020; Orassi et al., 2021; Pinheiro et al., 2021; Ruf et al., 2022; Luo et al., 2023; Yang et al., 2024). Therefore, a second local coordinate system (local_2_) was defined, as shown in Figure 5, following the convention described by Nelson to integrate their proposed loading conditions into the Baseline FE model (Nelson, 1986). The origin of this coordinate system was placed on the superior surface of the right condyle, centered on the articulating surface with the x-direction pointing to the left condylar center, the y-direction pointing posteriorly, and the z-direction pointing superiorly (Korioth and Hannam, 1994). These muscle forces can be weighted and scaled to match the desired subject of an FE study. Maximum muscle forces, fiber activations and direction cosines for each muscle, including superficial Masseter, deep Masseter, anterior Temporalis, medial Temporalis, posterior Temporalis, medial Pterygoid, and inferior lateral Pterygoid, are used to calculate force vectors (see Table 3; Supplementary Material Table SA3). These force vectors are coupled to reference points and kinematically coupled to the MAAs. The MAAs, were implemented as surface regions, defined anatomically based on literature and mapped onto the mandibular surface, therefore allowing the applied forces to be distributed (Liu et al., 2017; Ackland et al., 2018; Saini et al., 2020; Orassi et al., 2021). In addition, to replicate occlusion, the vertical displacement of the first left molar tooth was restricted (Orassi et al., 2021; Pinheiro et al., 2021). The condyles were assumed to be locked within the glenoid fossa and fixed in all DOFs to replicate the mechanical boundary conditions during peak occlusal loading (Lin and Su, 2020; Orassi et al., 2021).

**TABLE 3:** Comparison of all muscle forces used in the FE model. Table A reports forces in coordinate system local_2_, Table B and C in coordinate system local_1_.

(A) Resulting forces [N] for Baseline model (0) based on Nelson (Nelson, 1986; Korioth and Hannam, 1994)									
​	​	Balancing side			​	Working side			​
Muscle	​	F_x2_	F_y2_	F_z2_	F_total_	F_x2_	F_y2_	F_z2_	F_total_
Superficial Masseter	SM	−23.65	−47.87	100.99	114.23	28.38	−57.44	121.19	137.08
Deep Masseter	DM	−26.73	17.53	37.11	48.98	32.08	21.03	44.53	58.78
Medial Pterygoid	MP	50.97	−39.12	82.96	104.93	−71.36	−54.77	116.14	146.91
Anterior Temporalis	AT	−13.65	4.03	90.54	91.65	17.19	5.07	113.96	115.36
Medial Temporalis	MT	−14.22	32.03	53.61	64.05	14.01	31.55	52.81	63.09
Posterior Temporalis	PT	−6.13	25.21	13.98	29.47	9.28	38.14	21.14	44.58
Inferior lateral Pterygoid	ILP	27.40	−32.92	−7.57	43.49	−12.64	−15.19	−3.49	20.07

(B) Resulting forces [N] for sensitivity analysis from generic MMBS (Case 3-A)									
​	​	Balancing side			​	Working side			​
Muscle	​	F_x1_	F_y1_	F_z1_	F_total_	F_x1_	F_y1_	F_z1_	F_total_
Superficial Masseter	SM	50.75	−1.82	94.56	107.33	39.39	3.30	98.46	106.10
Deep Masseter	DAM	8.86	−0.44	12.83	15.60	6.32	0.82	13.48	14.91
	DPM	2.19	−0.88	6.36	6.78	1.07	0.66	6.91	7.02
Medial Pterygoid	MP	51.13	31.24	69.03	91.41	45.25	−31.96	81.04	98.17
Anterior Temporalis	AT	−9.24	−25.06	91.61	95.43	−26.27	24.39	114.60	120.08
Posterior Temporalis	PT	−4.18	−2.26	3.70	6.02	−25.27	10.81	20.52	34.30
Inferior lateral Pterygoid	ILP	10.73	8.08	−0.56	13.44	18.23	−15.66	0.59	24.04

(C) Resulting forces [N] for sensitivity analysis from scaled-generic MMBS (Case 3-B)									
​	​	Balancing side			​	Working side			​
Muscle	​	F_x1_	F_y1_	F_z1_	F_total_	F_x1_	F_y1_	F_z1_	F_total_
Superficial Masseter	SM	78.17	−13.18	100.56	128.05	49.85	19.95	88.59	103.59
Deep Masseter	DAM	13.54	−2.52	12.57	18.65	7.05	2.89	9.88	12.47
	DPM	2.44	−0.53	6.16	6.65	0.78	1.11	6.15	6.30
Medial Pterygoid	MP	39.19	23.81	39.00	60.20	78.23	−35.35	97.67	130.03
Anterior Temporalis	AT	−5.36	8.89	75.86	76.57	−28.22	20.38	153.09	157.00
Posterior Temporalis	PT	−28.17	−8.02	25.94	39.12	−28.14	13.04	25.69	40.27
Inferior lateral Pterygoid	ILP	1.90	1.20	−0.56	2.32	29.70	−19.60	−3.90	35.79

F_i1_ indicated the local_1_ coordinate system created based on the musculoskeletal multibody simulation (MMBS), F_i2_ indicated the local_2_ coordinate system created based on (Nelson, 1986; Korioth and Hannam, 1994). To compare muscle forces, the total Force (F_total_) for each muscle are presented.

##### 2.4.5.2 Case 1 - muscle attachment area (MAA)

The first sensitivity analysis examined the influence of different MAA shapes. As employed for the Baseline model, homogeneous material properties were also assumed for teeth, cortical and trabecular bone and the muscle forces proposed by Nelson for a left unilateral clenching on a first molar were applied to the mandible (Nelson, 1986; Korioth and Hannam, 1994). Unlike the Baseline model (0), to evaluate the isolated effect and importance of personalizing this input parameter, in case 1, we studied highly simplified MAAs of circular patches (Sass et al., 2025) with a radius of 3 mm.

##### 2.4.5.3 Case 2 - bone material properties (MP)

As it has been proven that the bone material model has a significant influence on the results of FE simulations (Xin et al., 2013; Hussein and Alruthea, 2021) in the second sensitivity study, the effect of different material properties should be evaluated. Similar to the Baseline model, the MAAs were anatomically defined, and the muscle forces proposed by Nelson for a left unilateral clenching on a first molar were applied to the mandible (Nelson, 1986; Korioth and Hannam, 1990). Unlike the Baseline model, where homogeneous material properties were applied in Case 2, the three most frequently used heterogeneous material models for the mandible bone (Soodmand and Becker et al., 2024) were compared (Table 2). While the material model proposed by Keyak et al. includes three different zones (cortical, transition, trabecular) (Case 2-A) and is developed based on the femur bone (Keyak et al., 1998), the Mimics default equation (Case 2-B) is the most used formula in dental FE models (Xin et al., 2013; Soodmand and Becker et al., 2024). The third case is a combined material model by Rho et al. for the cortical zone and O’Mahony et al. for the trabecular zone, with both materials specifically designed for the mandible bone (Rho et al., 1995; O'Mahony et al., 2000). If required, conversion from apparent to ash density in each formula was conducted using the correlation ρ_ash_ = 0.6 ρ_app_ (Schileo et al., 2008). To assign heterogeneous, isotropic material properties to the bone, the previously described in-house-developed MATLAB script for Abaqus was used (see Section 2.4).

##### 2.4.5.4 Case 3 - muscle forces (MF)

To study the influence of sequentially coupling the MMBS to the FE model to provide personalized loading conditions, we examined the sensitivity to muscle forces. Similar to the Baseline model, the MAAs were anatomically defined, and homogeneous material properties were assumed for teeth, cortical and trabecular bone. Unlike the Baseline (Case 0) model, in which the muscle forces from the Nelson study were applied, in case 3-A,B, muscle forces from generic and scaled-generic MMBS were applied, respectively.

**FIGURE 6:**
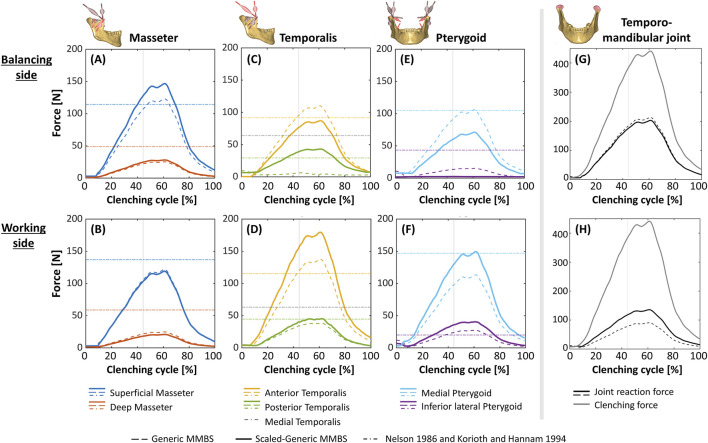
Different muscular loading conditions as input parameters to the finite element (FE) models in the present study. Comparison of three different muscular loading conditions for **(A,B)** Masseter, **(C,D)** Temporalis and **(E,F)** Pterygoid muscles on the balancing and working side of the mandible during a unilateral left-side clenching scenario. **(G,H)** illustrates joint reaction forces applied to the temporomandibular (TMJ) joint and clenching force on the first left molar. In all subfigures, dash-dot lines depict well-established and frequently used forces proposed by Nelson (1986), Korioth and Hannam (1994) for the maximum clenching force of 385 N and a single time-point, dashed lines show estimated time histories of muscle forces using the generic Point-on-the-plane model (Zee et al., 2007), and solid lines illustrate estimated time histories of forces obtained using a scaled-generic model that matches the medical image data of the subject of the present study. Vertical gray solid line at 44% of the clenching cycle shows the selected time point to read the muscle forces from the muscle force profiles calculated by the generic and scaled-generic musculoskeletal multibody model for the sensitivity study, where a clenching force of 385 N is applied, matching the clenching force of (Nelson, 1986; Korioth and Hannam, 1994).

To fairly compare case 3 with the Baseline in terms of muscle forces, we applied the same clenching force magnitude as in the Baseline (Case 0) model, which was 385 N (Nelson, 1986; Korioth and Hannam, 1994). As the MMBS simulation is based on experimentally captured motion and bite force data reaching up to 441 N (Zee et al., 2007), a specific time point within the simulation was selected at which the bite force equals 385 N. The corresponding muscle forces at this instant (occurring at 44% of the clenching cycle; see Figure 6) were then applied for comparison. This replicates the reaction forces resulting from the constraint, applied to the vertical displacement of the clenching tooth in the Baseline model. Next, the muscle forces from our generic MMBS, resulting in the same clenching force at 44% of the clenching cycle, were calculated for case 3-A. For case 3-B, the scaled-generic MMBS was used (Table 3). These calculated muscle forces are then applied, as an alternative to the muscle forces from Nelson (Nelson, 1986; Korioth and Hannam, 1994), to the Baseline model to build the FE models for case 3. In addition, since our MMBS, unlike the Baseline (0) model, does not include a medial Temporalis force element, the Temporalis muscle attachment area in the FE model in case 3 was halved and distributed to the anterior and posterior parts of the Temporalis (compare Baseline (0) and MF (3) cases in Figure 5). Additionally, our MMBS provides separate muscle elements for the anterior and posterior parts of the Masseter. Therefore, for case 3, the previously defined MAA in the Baseline (0) model was halved and assigned to the deep anterior Masseter and deep posterior Masseter.

In summary and for clarification, we generated eight FE models in total. First, the quasi-static MMBS-FE simulation framework developed in the present study (Figure 4), followed by sensitivity studies under static loading (Figure 5), including the Baseline (0) FE model, the sensitivity case 1 (MAA) model, the three sensitivity cases 2 (MP) models and the two sensitivity cases 3 (MF) models. As the static loading conditions proposed by Nelson were used for the sensitivity analysis, which is only available for one specific time point (Nelson, 1986; Korioth and Hannam, 1994), the modeling for the sensitivity study was static, while our proposed simulation framework was of the type quasi-static, based on the provided loading and boundary conditions by the MMBS over the time of clenching.

#### 2.4.6 Analysis of logarithmic strain and von Mises stress

The present study aims to estimate stress and strain values within mandibular bone at specific ROI with high clinical relevance, which are otherwise hard to evaluate with experiments (Orassi et al., 2021). The studied parameters include the magnitude and the distribution of the maximum von Mises stresses (S) and the maximum principal logarithmic strain (ε_1_, LE) (Ackland et al., 2017; Orassi et al., 2021), which was chosen to account for the geometrically nonlinear behavior (Hussein and Rabie, 2015). The proposed model analyzes the results over the full time period of the unilateral clenching as a quasi-static simulation. To focus more specifically on the studied parameter, the sensitivity analysis solely compares a single time step and therefore a static loading scenario of 385 N.

## 3 Results

### 3.1 Musculoskeletal multibody simulation

Figure 6 presents the time-dependent force profiles of the main closing and stabilizing muscles of the mandible during a unilateral left-side clenching scenario, including superficial and deep Masseter, anterior, medial, and posterior Temporalis, and medial and inferior lateral Pterygoid muscles on both working (left) and balancing (right) sides. These force profiles are 1) previously reported data by Nelson for a single time-point, where they presented corresponding muscle forces for a 385 N clenching force (Nelson, 1986; Korioth and Hannam, 1990), 2) calculated by the validated generic Point-on-the-plane mandible model (Zee et al., 2007) for varying clenching force between 0 and 441 N, and 3) calculated by the corresponding scaled-generic MMBS matching the medical image data of the subject of the present study.

All muscle and joint reaction forces on balancing and working sides, calculated by the MMBS, showed similar time-dependent profiles, with forces gradually increasing from the start of the clenching task, peaking around 60% of the clenching cycle, when the maximum bite force was applied, and a subsequent decrease toward a minimum at the end of the clenching.

For the Masseters (Figures 6A,B), the superficial head reached higher peak forces on the balancing side than on the working side for both the generic and scaled-generic MMBS, whereas the deep head showed smaller differences between the working and balancing sides (Figure 6). In contrast, the Temporalis (Figures 6C,D) and Pterygoid (Figures 6E,F) muscles generally exhibited peak forces on the working side higher than or similar to the balancing side in all three loading conditions, with the anterior Temporalis on the working side, with 179 N, followed by medial Pterygoid on the working side, with 149 N, showing the largest magnitudes. However, the TMJ joint reaction forces (Figures 6G,H) were higher on the balancing side than on the working side throughout the clenching, with calculated force magnitudes of 203 N by the scaled-generic MMBS and 213 N by the generic MMBS for the balancing side, and 136 N by the scaled-generic MMBS and 91 N by the generic MMBS for the working side.

Comparing the generic and scaled-generic MMBS, for the balancing side, the largest differences in muscle forces were observed in the posterior Temporalis and the inferior lateral Pterygoid. The posterior Temporalis reached a maximum force of 43 N during the clenching task in the scaled-generic model, whereas minimal activation of 4 N is calculated for the generic model. In contrast, the inferior lateral Pterygoid showed a maximum force of 15 N in the generic MMBS model but only 2 N in the scaled-generic model. On the working side, a consistent trend becomes visible, with the scaled-generic model producing higher peak forces than the generic model. The clearest effect of scaling in MMBS was observed for the inferior lateral Pterygoid with 32% or 13 N, for the medial Pterygoid with 24% or 36 N and for the anterior Temporalis 23% or 42 N, all showing increased peak forces for the scaled-generic model. A less prominent change can be seen for the working side superficial Masseter muscle, where an increase of only 2% or 3 N occurs due to scaling. Overall, the effect of scaling ranged from 3 N to 42 N for all studied muscles. In addition, scaling of the MMBS had a negligible effect of 5% or 10 N on the TMJ reaction force prediction on the balancing side. However, it caused a considerable increase of 33% or 44 N on the working side TMJ.

Figure 6 also highlights the clear difference between the static loading condition proposed by Nelson (Nelson, 1986; Korioth and Hannam, 1990) for the subject of their study and muscle forces calculated for the subject of the present study using the MMBS model. To enable reproducibility for subsequent studies, the applied clenching force profile on the first left molar, the boundary conditions including the displacement of the first molar and condyles, as shown in Figure 4A, and the muscle forces as loading conditions, calculated using the scaled-generic MMBS, are presented in the Supplementary Material Table SA1.

### 3.2 Finite element analysis using the presented simulation framework

**FIGURE 7:**
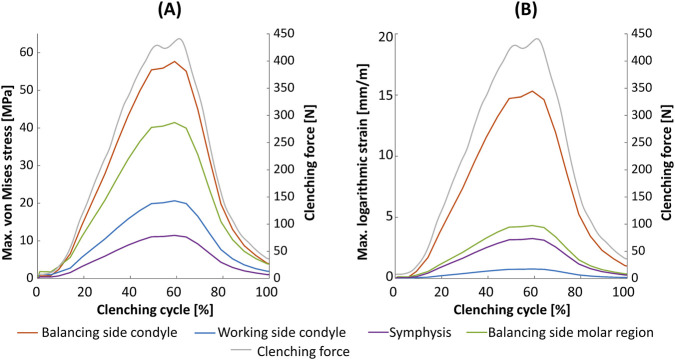
Studied mechanical response of the bone using the proposed quasi-static simulation framework of a sequentially coupled MMBS-FE model during unilateral left-side clenching in the present study. **(A)** Von Mises stress and **(B)** logarithmic strain under unilateral left-side clenching scenario in different bone regions of interest, including balancing/working side condyle, symphysis, and balancing side molar regions.

Our quasi-static FE mandible model comprises 356,951 quadratic tetrahedral elements (C3D10) with an element size of 1.8 mm and a mesh refinement of 0.6 mm in the working side molar region. Figure 7 shows the maximum von Mises stress and logarithmic strain values in the ROIs during the unilateral left-side clenching scenario, calculated over time by the presented framework. Both values closely follow the clenching force profile (Figure 7), peaking at similar time points, rising from zero, peaking near the maximum bite force (441 N) at 6.1 s, and then returning to the minimum value at the end of the clenching.

The balancing side condyle region exhibited the largest responses among all ROIs, followed by the balancing side molar region and the working side condyle or symphysis, with all closely matching the clenching force profile (Figure 7). The balancing side condyle experienced the highest levels of maximum stress and strain, with stress higher than in other ROIs, with a difference in maximum value of 58 MPa compared to 41 MPa in the balancing side molar region. The stress in the working side condyle was considerably lower, and the symphysis was minimal. A similar trend is observed for the maximum logarithmic strain, indicating a balancing-side dominant pattern for the unilateral left-side clenching scenario. Although the strain was nearly negligible for the working side condyle, this ROI experienced higher stresses than the symphysis.

### 3.3 Sensitivity analysis on model input parameters

**FIGURE 8:**
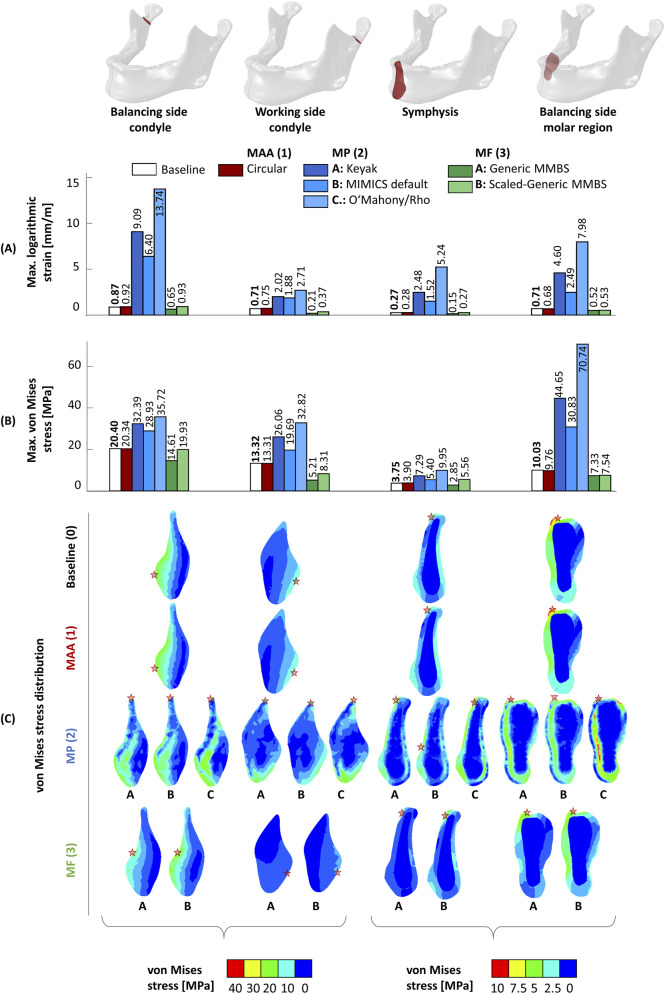
Sensitivity analysis on the effect of input parameters for the sequentially coupled MMBS-FE model on the simulation results. Case 1: effect of muscle attachment area (MAA), case 2: material properties (MP), and case 3: muscle forces (MF) as loading conditions are shown on the **(A)** logarithmic strain, **(B)** the von Mises stress, and **(C)** stress distribution under unilateral left-side clenching scenario in different bone regions of interest, including balancing/working side condyle, symphysis, and balancing side molar regions. The red star shows the location of the max von Mises stress for each case.

The sensitivity analysis is performed under static conditions for a unilateral left-side clenching load of 385 N. Figure 8 illustrates the sensitivity of the FE model outcomes to variations in selected input parameters, including the effect of muscle attachment area (MAA), material properties (MP) of the bone, and muscle forces (MF). The analysis compares the resulting maximum logarithmic strain (Figure 8A), maximum von Mises stress (Figure 8B), and the corresponding spatial stress distributions (Figure 8C) across four regions of interest (ROIs) at the balancing and working side condyles, the symphysis, and the balancing side molar.

Overall, variations in bone material properties as input parameters produced the largest deviations from the Baseline model, particularly in stress values in the balancing-side molar region and strains in the balancing-side condyle. The effect of the muscle attachment area was, however, relatively negligible at almost all ROIs, while changes in applied muscle forces led to smaller changes than the MP, but still noticeable effects, primarily on stress magnitudes.

Regarding the effect of input parameters at each ROI in more detail, the sensitivity cases influenced the stress magnitudes at most at the balancing side molar region, with an average of 202% or 20.27 MPa over all sensitivity cases, while the influence was minimal on the balancing side condyle, with an average of 34% or 7.01 MPa over all sensitivity cases (Table 4B). The strain values were most affected at the symphysis, with an average of 534% or 1.43 mm/m increase over all sensitivity cases compared to the Baseline case. The smallest but still considerable effect was observed in the working-side condyle region, with an average of 126% or 0.89 mm/m increase over all sensitivity cases compared to the Baseline case (Table 4A). Considering the absolute magnitudes of the mechanical responses, the working side condyle showed the lowest strain levels, while the symphysis showed the smallest stress magnitudes across all cases. The balancing side condyle consistently showed the highest mechanical response among all ROIs, with von Mises stress exceeding that of the other ROIs, except for the balancing side molar region in several MP variation cases (2-A to 2-C), which exhibited higher stresses with peak von Mises stress exceeding 50 MPa. The Symphysis region exhibited comparatively low stress levels, ranging below 10 MPa, across all simulations.

**TABLE 4:** Sensitivity analysis of the effects of model input parameters on the mechanical response of bone.

(A) Max logarithmic Strain [mm/m]									
Region of interest	​	Baseline Case 0	Case 1	Case 2-A	Case 2-B	Case 2-C	Case 3-A	Case 3-B	Average per region
Balancing side condyle		0.87	0.92	9.09	6.40	13.74	0.65	0.93	​
Relative changes [%]		​	**5%**	**942%**	**634%**	**1,475%**	−25%	**7%**	**515%**
Absolute changes [mm/m]		​	0.04	8.22	5.53	12.87	−0.22	0.06	4.49 ± 5.34
Working side condyle		0.71	0.75	2.02	1.88	2.71	0.21	0.37	​
Relative Absolute chabges [mm/m]		​	**6%**	**185%**	**166%**	**283%**	−71%	−48%	**127%**
Absolute changes [mm/m]		​	0.04	1.31	1.17	2.00	−0.50	−0.34	0.89 ± 0.73
Symphysis		0.27	0.28	2.48	1.52	5.24	0.15	0.27	​
Relative changes [%]		​	**6%**	**828%**	**468%**	**1859%**	−44%	**2%**	**534%**
Absolute changes [mm/m]		​	0.01	2.22	1.25	4.97	−0.12	0.01	1.43 ± 1.95
Balancing side molar region		0.71	0.68	4.60	2.49	7.98	0.52	0.53	​
Relative changes [%]		​	**−3%**	**551%**	**252%**	**1,030%**	−27%	−25%	**314%**
Absolute changes [mm/m]		​	−0.02	3.89	1.78	7.27	−0.19	−0.18	2.22 ± 2.87
Average per sensitivity case	Relative changes [%]	​	**5%**	**627%**	**380%**	**1,162%**	42%	21%	​
	Absolute changes [mm/m]	​	0.03 ± 0.01	3.91 ± 3.07	2.43 ± 2.08	6.78 ± 4.50	0.26 ± 0.17	0.15 ± 0.15	​
Average per parameter	Relative changes [%]	​	**5%**	723%			31%		​
	Absolute changes [mm/m]	​	0.03 ± 0.01	4.37 ± 3.61			0.20 ± 0.16		​

(B) Max von Mises Stress [MPa]									
Region of interest	​	Baseline Case 0	Case 1	Case 2-A	Case 2-B	Case 2-C	Case 3-A	Case 3-B	Average per region
Balancing side condyle		20.40	20.34	32.39	28.93	35.72	14.61	19.93	​
Relative changes [%]		​	**0%**	59%	42%	75%	−28%	**−2%**	34%
Absolute changes [MPa]		​	−0.06	11.99	8.53	15.32	−5.79	−0.47	7.01 ± 6.14
Working side condyle		13.32	13.31	26.06	19.69	32.82	5.21	8.31	​
Relative changes [%]		​	**0%**	96%	48%	**146%**	−61%	−38%	65%
Absolute changes [MPa]		​	−0.01	12.74	6.37	19.50	−8.11	−5.01	8.62 ± 6.75
Symphysis		3.75	3.90	7.29	5.40	9.95	2.85	5.56	​
Relative changes [%]		​	**4%**	94%	44%	**166%**	−24%	48%	63%
Absolute changes [MPa]		​	0.15	3.54	1.66	6.20	−0.90	1.81	2.38 ± 2.19
Balancing side molar region		10.03	9.76	44.65	30.83	70.74	7.33	7.54	​
Relative changes [%]		​	**−3%**	**345%**	**207%**	**605%**	−27%	−25%	**202%**
Absolute changes [MPa]		​	−0.27	34.62	20.80	60.71	−2.70	−2.49	20.27 ± 23.94
Average per sensitivity case	Relative changes [%]	​	**2%**	**149%**	85%	**248%**	35%	28%	​
	Absolute changes [MPa]	​	0.12 ± 0.11	15.72 ± 13.27	9.34 ± 8.16	25.43 ± 24.16	4.37 ± 3.21	2.45 ± 1.91	​
Average per parameter	Relative changes [%]	​	**2%**	161%			32%		​
	Absolute changes [MPa]	​	0.12 ± 0.11	16.83 ± 16.53			3.41 ± 2.65		​

The sensitivity analysis is performed under a static unilateral left-side clenching condition of 385 N. **A.** Maximum von Mises stress [MPa] and **B.** Maximum logarithmic strain [mm/m]. Relative and absolute changes compared to the Baseline model (positive indicates an increase, negative a decrease) are calculated for all ROI (balancing/working side condyle symphysis and balancing side molar region) and sensitivity cases (1 = Muscle Attachment Areas, 2 = Material Properties (2-A = Keyak et al., 2-B = MIMICS equation, 2-C = O’Mahony and Rho), 3 = Muscle Forces (3-A = generic MMBS, 3-B = scaled-generic MMBS)). Also, average relative and absolute changes with their standard deviation are calculated for each region and sensitivity case, respectively. Changes smaller than 10% are highlighted in green, and changes larger than 100% are highlighted in red.

Regarding the level of influence of each input parameter on the stress magnitudes, changing the material properties showed the highest effect on the outcomes of the FE analysis, with an average of 161% or 16.83 MPa across all ROIs and the three cases of MP (2-A, 2-B, 2-C) (Table 4B). The maximum change was made by varying the material properties of the mandible bone from homogeneous in the Baseline to heterogeneous, as defined in case 2-C by Rho et al. and O’Mahony et al. (Rho et al., 1995; O'Mahony et al., 2000) at the balancing side molar region (Figure 8B) with an increase of 605% or 60.71 MPa in the estimated max von Mises stresses. However, negligible changes were observed when reshaping the muscle attachment area (Case 1) from anatomical shapes in the Baseline to a simplified circular shape in the MAA case, with an average change of 2% or 0.12 MPa across all ROIs.

In terms of the effect on the strain values, the change in results was more pronounced, with an average increase of 723% or 4.37 mm/m across all ROIs and cases 2-A, 2-B and 2-C, (Table 4A), which was due to changes in material properties. The maximum strain change was made by varying the material properties from homogeneous to heterogeneous material properties as defined in case 2-C at the symphysis (Figure 8A) with an increase of 1859% or 4.97 mm/m. Variations in applied muscle forces (Cases 3-A and 3-B) produced proportional changes in both stress and strain, with the largest impact observed at the working side condyle for case 3-A, with a decrease of 61% or 8.11 MPa in stress and 71% or 0.5 mm/m in strain (Table 4). In general, muscle force either decreased the mechanical response or remained relatively unchanged, except for case 3-B in Symphysis, which increased the stress magnitude by 48% or 1.81 MPa.

Figure 8C illustrates the resulting stress distribution contours, which show wider high-stress zones at the balancing-side molar region due to material variations and a shift in the maximum stress location, particularly in the condyles, where it moved antero-superiorly (see Figure 4B for the directions). As for the effect of the muscle forces (Cases 3-A and 3-B), the stress distribution pattern and the location of maximum stress remained nearly close to the Baseline.

## 4 Discussion

In the present study, we developed a computational framework that sequentially coupled a scaled-generic musculoskeletal multibody simulation (MMBS) with a subject-specific finite element (FE) model, thereby bridging organ- and tissue-level analyses to investigate the biomechanics of the human masticatory system. The framework successfully predicted mandible biomechanics under a unilateral clenching task. The importance of scaled-generic MMBS was demonstrated by the substantial deviation between the muscle forces reported in the literature and those calculated by scaled-generic MMBS. Additionally, this study contributed to a systematic sensitivity analysis of the key model input parameters to the FE models of the masticatory system. We investigated how variations in input parameters influence the outcomes of the FE simulation and found that bone material properties (MP) play the most influential role, followed by minor effects from applied muscle forces (MF) as a function of the loading conditions, and negligible effects from the muscle attachment area (MAA). Among all studied regions of interest (ROIs), the balancing-side condyle shows the highest response in the main sequentially coupled MMBS-FE simulation (Figure 7), while the sensitivity analysis indicated that peak von Mises stress can shift to the balancing-side molar region under certain parameter settings (Figure 8B; Table 4B). Nevertheless, the highest stress peaks remained on the balancing side, confirming increased mechanical loading during unilateral clenching.

### 4.1 Loading and boundary conditions estimated by musculoskeletal multibody simulation

The commonly used muscular loading conditions presented by (Nelson, 1986; Korioth and Hannam, 1994), as depicted in Figure 6, were derived under static equilibrium for a specific biting configuration of the masticatory system, at a single moment of applying a force of 385 N. In contrast, integrating muscle forces from MMBS during unilateral clenching within our simulation framework enabled consideration of the time-dependent, dynamic coordination of multiple muscle groups, which is required for generating realistic loading in the FE model. Static FE analysis cannot reproduce the effects of relative activation timing and direction of muscles, the so-called temporal coordination, and therefore may potentially lead to inaccurate force distribution and stress predictions across the mandible surface. In addition, the proposed integration of moving boundary conditions and displacement in an FE model enables a detailed analysis of mandibular biomechanics and can be especially useful for more dynamic tasks such as chewing and protrusion.

Our MMBS predicted the main contributors to bite force generation (Figure 6), in agreement with the previous computational studies, to be the superficial Masseter, anterior Temporalis and medial Pterygoid (Ackland et al., 2017; Woodford et al., 2024). Also, the general working side dominance in muscle force generation is consistent with the findings of previous simulations (Woodford et al., 2024). Additionally, we observed that the superior lateral Pterygoids, as well as the suprahyoid muscles, contributed very little to clenching, which is in full agreement with the literature (Ackland et al., 2017; Woodford et al., 2024). During unilateral clenching, the maximum predicted muscle force through the simulated clenching cycle was exerted by all muscles at the time point at which the maximum clenching force was applied by the subject (Figure 6) (Belser and Hannam, 1986). Early contractions of the anterior Temporalis, especially on the working side, initiate mandible elevation toward the clenching position (Figures 6C,D) (Aldana et al., 2011; Thomas, 2023), followed by activation of the Masseters (Figures 6A,B).

In addition, the present MMBS is based on a model of the human masticatory system from Anybody Modeling System™, previously validated against EMG measurements for the superficial Masseter, anterior Temporalis, and medial Pterygoid, showing good agreement in timing and amplitude during clenching tasks (Zee et al., 2007). This supports the plausibility of the overall muscle activation patterns predicted in our study. Nevertheless, we found relatively low force contributions in the deep Masseter muscles (Figures 6A,B), which is consistent with previous musculoskeletal models of the masticatory system (Ackland et al., 2017; Woodford et al., 2024). However, further experimental measurements are required to confirm this. Fine-wire EMG studies reported equal or greater activation in the deep Masseter than in the superficial Masseter during intercuspal clenching, indicating that deep Masseter loading is likely under-estimated in current recruitment strategies (Belser and Hannam, 1986; van Eijden et al., 1993). This observed deviation highlights where future work should focus, particularly on the muscle recruitment cost function in static optimization, since predicted muscle forces are known to be sensitive to the choice of recruitment criterion (Monaco et al., 2011; Michaud et al., 2021).

The static muscular loading conditions proposed by Nelson were derived for the specific subject anatomy of that study (Nelson, 1986) and therefore cannot replicate the individual characteristics of subjects in other studies, which is a known source of modeling error (Iwasaki et al., 2004; Angst et al., 2024). Furthermore, Figure 8 clearly illustrates the substantial impact of scaling a generic MMBS on predicted muscular loading conditions in comparison to the generic MMBS, resulting in higher estimated force magnitudes by up to 32% or 42 N, while maintaining similar timing and coordination among muscle groups. In particular, scaling had a large effect on Temporalis and Pterygoid forces, but a minimal influence on Masseter. As geometric scaling affects the muscle moment arms and joint coordinates, the Temporalis and Pterygoid, with longer and more oblique fibers, are more affected by geometric scaling, whereas the line of action and consequently the muscle forces of the Masseter show less sensitivity to scaling.

### 4.2 Evaluation of finite element and sensitivity analysis results

The synchronized variation between force and resulting bone mechanical responses (Figures 6, 7) indicates an instant response of the mandible bone, proportional to the externally applied clenching force. Also, the absence of a noticeable time shift between the applied force and the mechanical bone response (Figure 7) confirms the assumption of negligible inertial effects in the quasi-static FE analysis and that the bone maintained near-equilibrium conditions throughout the clenching task.

The largest von Mises stress and logarithmic strain magnitudes among all ROIs were observed in the balancing-side condyle (Figure 7), indicating greater load transfer and geometric stress concentration at this site and suggesting that this region experiences higher reaction forces. This is consistent with our MMBS results, as shown in Figure 6, and by previous biomechanical models, in which balancing-side TMJ can exceed the working side in joint loading (Ferrario and Sforza, 1994; Woodford et al., 2024). Following this, the balancing side molar region showed the largest mechanical responses. This balancing side-dominant response pattern explains the load transfer across the mandibular arch during unilateral clenching, where muscle forces on the working side compress the occlusal region, while reaction forces at the balancing side generate higher stresses (Korioth and Hannam, 1990; Ferrario and Sforza, 1994; Throckmorton, 2000).

Although the strain magnitudes were small on the working-side condyle, this ROI exhibited higher stress than the symphysis. This clearly suggests that the applied deformation amplitude alone is not the only influencing factor in the stress distribution within the bone, but localized stiffness and geometry also play a considerable role. This is further supported by the sensitivity analysis, which showed that changing from homogeneous to heterogeneous material or altering the density-modulus relationship (Case 2) had the strongest influence on the biomechanical outcome parameters. Therefore, bone material properties were identified as the most influential input parameter of the FE model (Figure 8). Previous studies similarly emphasize that incorporating precise material properties is essential for subject-specific FE modeling (Poelert et al., 2013; Oefner et al., 2021). However, Soodmand and Becker et al. reported that no consensus exists regarding the reliability of density-modulus relationships for heterogeneous mandibular bone assignment and that several frequently used relationships were originally developed for other anatomical sites, such as the femur, tibia, or pelvis, leading to markedly different modulus ranges (Soodmand and Becker et al., 2024). Consistent with this, Hussein and Alruthea demonstrated substantial variability in FE results when different density-modulus relationships were used (Hussein and Alruthea, 2021). Because the present study did not include direct experimental validation, it cannot identify a single best material model. Instead, it demonstrates that the selected material law substantially affects the predicted field variables and should therefore be prioritized in future validation studies. This is consistent with comparative studies of other anatomical structures, e.g., the femur (Schileo et al., 2008), where systematic comparisons would be beneficial to identify the most consistent material model. A less influential, but still considerable, effect of the model input parameters was identified as muscle forces, with changes of up to 71% in the sensitivity analysis (Figure 8; Table 4). If building scaled-generic or patient-specific MMBS is not feasible, we recommend using at least a generic MMBS to define the loading and the boundary conditions. This is particularly important when the region of interest is located close to the muscle attachment sites, e.g., at the working side condyle (Table 4) and in pathological cases such as patients with segmental mandibular resection (Ruf et al., 2026), in which muscle coordination may differ substantially from that of healthy subjects and cannot be represented by simply scaling down the muscles strength. In the ideal case, when anthropometric and geometric data are available from the subject, scaled-generic or patient-specific MMBS should be preferred, as they can provide more realistic muscle force magnitudes together with more accurate muscle moment arms and line of actions compared to the static data from the literature for another healthy subject (Nelson, 1986; Korioth and Hannam, 1994) and line of actions compared to the static data from the literature for another healthy subject. In contrast, the sensitivity analysis showed that even an extreme change in the definition of the muscle attachment area in the FE model, from anatomically shaped to circular patches, introduced an effect of less than 6%. Therefore, we recommend that an approximately anatomical representation of the attachment area is sufficient for an acceptable estimation of the mandibular stress and strain for the investigated load case.

In addition to assessing the overall influence of input parameters, the sensitivity analysis also revealed ROI-dependent differences. Although bone material properties were the dominant source of variation in all ROIs, their effect was not spatially uniform. The largest relative strain changes were observed at the symphysis, whereas the largest absolute strain changes occurred at the balancing-side condyle. For von Mises stress, the balancing-side molar region showed the greatest relative and absolute variation, while the condylar regions exhibit both relatively high baseline loading and substantial sensitivity to changes in material assignment (Figure 8; Table 4). This distinction is important because regions with low magnitudes of response in the baseline model, such as symphysis, may exhibit large relative changes without showing the largest absolute mechanical differences. Therefore, when interpreting the sensitivity results, both relative and absolute measures should be considered. From a practical perspective, our present findings (Figure 8; Table 4) do not support the use of simplified material modeling or muscle loading conditions for any ROIs when stress- or strain-based outcomes are of interest. The findings suggest that more realistic muscle forces and the choice of material properties are particularly critical when the condyles or posterior mandible are the primary regions of interest, for example, in studies of fracture risk, fixation stability, or TMJ implant biomechanics.

To further evaluate the plausibility of the proposed simulation framework, we also placed the FE analysis outcomes in the context of existing experimental measurements of bone mechanical responses as follows. A challenge of experimental studies is the non-invasive measurement of internal stresses within the mandible. Therefore, experimental studies typically only assess the deformation using optical tracking systems and digital image correlation, or they measure surface strain using strain gauges attached to the bone (Gröning et al., 2009; Ramos et al., 2011; Mesnard and Ramos, 2016; Ingawale et al., 2022; Banerjee et al., 2023). Ingawale et al. conducted experiments on human cadaveric mandibles and reported surface strain patterns under controlled cyclic loading of 140 N–200 N for five mandibles (Ingawale et al., 2022). Surface strains in the condyles showed the highest absolute values of strain compared to other regions, ranging from 0.003 mm/m to 0.024 mm/m. The molar region exhibited a low value of less than 0.001 mm/m. These results show a high difference to the reported values in our framework. However, their study design differs substantially from physiological conditions and consequently from our FE model, which complicates comparisons. On the other hand, Ramos et al. presented a more physiologically relevant setup by applying the Masseter and Temporalis muscle forces on both sides using strings and constraining one molar to represent unilateral clenching (Ramos et al., 2011). Unlike our proposed framework, their numerical-experimental study used a synthetic mandible and therefore applied small muscle loadings of up to 12 N, focusing on surface strains at different ROIs. They report maximum principal surface strains of around 0.8 mm/m in the condyle region on the working side. These results are consistent with our results, which showed a maximum strain peak of 1 mm/m.

As there is a lack of experimental data suitable for validating mandible models (Chang et al., 2018; Merema et al., 2021), we used well-established previous computational studies to evaluate the predicted outcomes for the studied ROIs. Pinheiro et al. investigated the biomechanical behavior of the mandible following temporomandibular joint replacement under left unilateral clenching tasks of 455.5 N and with a complex modelling of the joint, including the fossa and the disk (Pinheiro et al., 2021). They predicted an average principal strain of 0.26 mm/m, reaching values of around 1.5 mm/m for both condyles and 0.5 mm/m in the balancing-side molar region. This is comparable to the results of our proposed framework, 1 mm/m for the working-side condyle, but underestimates our results for the balancing-side condyle and balancing-side molar region (Figure 6). Lin and Su performed a FE analysis to investigate how different occlusion conditions influence the mechanical response of dental implants placed at various positions in the mandible, and therefore also investigated the stress distributions of the bone surface (Lin and Su, 2020). Their simulations examined a right-side unilateral clenching scenario, following a similar model set-up to our simplified Baseline model. The highest von Mises stress was observed in the balancing-side condyle region, at around 35 MPa, which is in a similar range to our findings, with 45 MPa. They also report von Mises stress of 15 MPa for the symphysis and around 20 MPa for the working side condyle. Our proposed frameworks simulate the maximum von Mises stress in the respective ROIs, 10 MPa and 20 MPa.

Taken together, these results demonstrate that the sequentially coupled MMBS-FE framework reproduces plausible muscle loading and mandibular bone mechanics. It captures both the temporal coordination of loadings and the characteristic side-specific asymmetry of stress transfer during unilateral clenching.

### 4.3 Limitations and future works

While many previous MMBS and FE studies employ simplifications, the present study addresses several of these limitations by using a personalized mandible geometry, heterogeneous material definitions, and a sequentially coupled MMBS-FE framework. Nevertheless, certain limitations remain, leading to further research questions that should be addressed in future work.

Due to the anonymization of the CT images of the subject of this study and the lack of donor-specific information, further personalization of the generic MMBS was not feasible. This resulted in three major simplifications: the muscle-tendon parameters, e.g., optimal fiber length and maximum force capacity, could not be individually adjusted, and no personalized motion and bite-force data could be obtained from the same subject for this study. Moreover, the lack of personalized muscle-tendon parameters is associated with several limitations. In MMBS models of the human TMJ, muscles are typically represented by force vectors that connect approximate muscle origin and insertion centroids (centroid-to-centroid force vectors) (She et al., 2018). This simplification assumes equivalent moment arms and muscle lengths for large groups of fibers within a muscle, even with complex geometry, and may result in inaccurate estimates of muscle force and joint loading. In addition, it reduces the anatomical detail of muscles with broad attachment areas by representing them with a limited number of actuator elements. In the present model, for example, the Temporalis was represented by anterior and posterior heads rather than by its full regional fiber architecture. Additionally, it neglects muscle wrapping, changes in fiber direction, and volume effects (Blemker and Delp, 2005). Future studies should investigate the personalization of the muscle-tendon unit based on MR images and EMG data (Manns et al., 1979). The second simplification, lack of subject-specific motion data, introduces a mismatch between kinematic and anatomical datasets (Zee et al., 2007), leading to inaccuracies. Further simplifications of the MMBS include the use of a point-on-the-plane constraint to represent condylar contact and the TMJ joint articulation. As the mandibular fossa forms a complex joint surface with both translational and rotational motion, this simplified constraint does not accurately reproduce the true TMJ kinematics. A force-dependent kinematic framework could address this limitation by allowing the joint motion to emerge from the balance between muscle forces and joint reaction forces, leading to more physiologically realistic TMJ behavior (Skipper Andersen et al., 2017).

From the FE perspective, several general simplifications were necessary: The periodontal ligament (PDL) was not included, and the tooth-bone interfaces were assumed to be perfectly osseointegrated. This represents an idealization that does not capture the clinical variability in biological integration. However, this choice was made because the studied ROIs are sufficiently far from the teeth, and therefore, the effect of this simplification should be negligible. In addition, the mandible bone was modeled as an isotropic and linearly elastic material with spatially varying elastic modulus, although bone tissue exhibits anisotropic mechanical behavior (Soodmand and Becker et al., 2024). Furthermore, trabecular bone was modeled as a homogenized solid continuum rather than an explicit porous network, following the standard in the literature, and to reduce computational complexity. Regarding the area of muscle attachments, no relevant differences were observed in the sensitivity analysis results; however, their positions were not personalized, which would require subject-specific MRI data or image-based reconstruction from CT (Neumann et al., 2024). Furthermore, the analyzed ROIs were not located within the MAAs, potentially influencing the results and effects of MAA variations. Additionally, only the primary masticatory muscles were included in the FE model, while the suprahyoid and infrahyoid muscles were excluded, which are necessary for investigating more complex tasks. Although their inclusion would be feasible within the modeling framework, their contribution to mandibular loading during clenching is considered negligible, making this a reasonable simplification to reduce computational effort. Moreover, the present analysis was performed under quasi-static conditions, neglecting time-dependent behaviors such as fatigue, wear, or viscoelastic responses, particularly for more complicated dynamic movements, e.g., chewing.

In the present analysis, only the maximum von Mises stress and strain values, as well as the spatial distribution of the von Mises stress, were evaluated. While this approach provides valuable insight into local mechanical behavior, it captures only a limited portion of the mandible biomechanical response. Future work could include the evaluation of strain energy density, principal strain orientation, and stress gradients to better characterize the local load transfer mechanisms (Liu et al., 2017; Li et al., 2023).

It should be noted that the framework was used to simulate a single healthy subject and a unilateral clenching task. As a result, inter-individual variability in anatomy, material properties, and muscle recruitment, as well as task-dependent differences in mandibular loading, were not addressed. Consequently, the present work should be regarded as a proof-of-concept demonstration of the sequentially coupled MMBS-FE framework, and the biomechanical findings and interpretations should not be generalized. Broader biomechanical conclusions will require subject-specific cohorts that also include disease-specific factors such as altered anatomy, muscle dysfunction or changes in condylar movements, as well as motion data for a wider range of functional scenarios, including chewing, protrusion, or laterotrusion (Skipper Andersen et al., 2017; Shigemitsu et al., 2020; Becker et al., 2025).

In general, the use of the presented sequentially coupled MMBS-FE framework represents a methodological step, providing a more realistic loading representation than purely artificial axial or inclined loading conditions frequently reported in the literature (Merema et al., 2021). Nevertheless, the workflow remains one-way as there is no feedback from FE analysis to MMBS. In addition, the applied loading conditions cannot fully reproduce the complex mechanical environment present *in vivo*, and therefore, an experimental validation would be necessary. As the analyzed mandible was not available for biomechanical testing, no experimental validation could be performed, indicating validation as a future research question.

## 5 Conclusion

In conclusion, this study successfully presented a computational framework that sequentially couples a scaled-generic MMBS with a subject-specific FE model to analyze unilateral clenching under more realistic loading and boundary conditions. The MMBS provides time-dependent muscle forces and mandibular landmark displacements, which drive a quasi-static FE analysis of the mandible. The proposed framework addresses several limitations of previous FE studies and highlights the importance of various modelling parameters. The sensitivity analysis identified bone material properties as the most influential input parameter, followed by muscle forces, with muscle attachment area having only a minor effect. These results emphasize the need to prioritize realistic material characterization and loading conditions in subject-specific mandibular FE models. The present study includes limitations and should be regarded as a proof-of-concept MMBS-FE framework. Nevertheless, it provides a reproducible basis for computational investigations of the masticatory system and offers guidance and recommendations for improving model reliability, preoperative planning, and implant design in the craniomaxillofacial field.

## Data Availability

The original contributions presented in the study are included in the article/Supplementary Material, further inquiries can be directed to the corresponding author.
