# Supplementary material for: Sequentially coupled musculoskeletal multibody and finite element simulation for biomechanical modeling of the human masticatory system
Source: Front Bioeng Biotechnol. 2026 Jun 23;14:1809299. doi: 10.3389/fbioe.2026.1809299 (PMC13337648; doi:10.3389/fbioe.2026.1809299)
Supplement: Supplementary file 1 [file Supplementaryfile1.pdf]

1 Supplementary Tables

Supplementary Table A1 | Exported parameters from scaled musculo-skeletal multibody simulation for unilateral left side clenching used as input for proposed FE model. Exemplary muscle forces (AT = anterior Temporalis, MP = medial Pterygoid, SM = superficial Masseter), bite force and displacement are shown with their x,y,z components.

	Bite force [N]			Exemplary muscle forces [N]																	
				Working side									Balancing side								
				AT			MP			SM			AT			MP			SM		
**Time**	F_x	F_y	F_z	F_x	F_y	F_z	F_x	F_y	F_z	F_x	F_y	F_z	F_x	F_y	F_z	F_x	F_y	F_z	F_x	F_y	F_z
**0.5**	−2	0	−10	−1	0	4	4	−2	5	2	1	3	0	0	0	5	3	5	2	0	2
**1.0**	−5	−1	−26	−2	2	12	8	−3	10	3	1	4	0	0	4	5	3	5	5	−1	7
**1.5**	−13	−2	−65	−5	4	28	15	−7	19	9	4	16	−1	2	13	7	4	7	14	−2	18
**2.0**	−24	−4	−122	−9	7	50	27	−12	34	17	6	29	−2	3	25	13	8	13	26	−4	33
**2.5**	−34	−6	−176	−13	9	72	38	−17	47	24	9	42	−3	4	36	19	11	19	37	−6	47
**3.0**	−43	−8	−225	−17	12	91	48	−22	59	30	12	53	−3	5	46	24	14	24	47	−8	60
**3.5**	−54	−10	−287	−21	15	116	61	−27	75	38	15	67	−4	7	58	30	18	30	59	−10	76
**4.0**	−63	−11	−337	−25	18	136	71	−32	88	45	18	79	−5	8	68	35	21	35	70	−12	90
**4.5**	−70	−13	−383	−29	21	155	79	−36	99	50	20	90	−5	9	77	40	24	39	79	−13	102
**5.0**	−77	−14	−418	−31	22	169	86	−39	108	55	22	98	−6	10	84	44	27	44	86	−15	111
**5.5**	−76	−14	−416	−31	22	168	86	−39	107	55	22	98	−6	10	83	44	27	44	85	−15	111
**6.0**	−79	−15	−432	−33	23	175	89	−40	111	57	23	102	−6	10	86	46	28	46	88	−15	115
**6.5**	−74	−13	−397	−30	21	161	83	−37	103	52	21	93	−6	9	80	42	25	42	82	−14	106
**7.0**	−61	−11	−325	−25	18	132	68	−31	86	43	17	77	−5	8	66	35	21	35	67	−11	87
**7.5**	−44	−8	−226	−17	12	91	49	−22	61	30	12	54	−3	5	46	25	15	24	47	−8	61
**8.0**	−28	−5	−142	−11	8	58	31	−14	39	19	8	34	−2	3	29	16	9	16	30	−5	39
**8.5**	−19	−3	−98	−8	5	41	22	−10	28	13	5	24	−1	2	20	11	7	11	21	−4	27
**9.0**	−14	−2	−69	−5	4	29	16	−7	20	10	4	17	−1	2	14	8	5	8	15	−3	19
**9.5**	−10	−2	−48	−4	3	21	12	−5	15	7	3	12	−1	1	10	5	3	5	11	−2	14
**10.0**	−7	−1	−35	−3	2	16	9	−4	12	5	2	9	−1	1	7	5	3	5	8	−1	10

	Displacement per Time step [mm]								
	Working side TMJ			Balancing side TMJ			Molar		
**Time**	X	Y	Z	X	Y	Z	X	Y	Z
**0.5**									
**1.0**	−0.20	0	0.12	−0.17	0.00	0.10	−0.16	0.02	0.14
**1.5**	−0.41	0	0.24	−0.28	−0.01	0.16	−0.19	0.09	0.39
**2.0**	−0.13	0	0.07	−0.20	0.01	0.12	0.21	−0.07	0.46
**2.5**	−0.09	0	0.05	−0.14	0.00	0.08	0.07	−0.04	0.25
**3.0**	−0.06	0	0.03	−0.07	0.00	0.04	0.12	−0.01	0.22
**3.5**	−0.01	0	0.01	−0.01	0.00	0.01	0.14	0.00	0.16
**4.0**	−0.08	0	0.05	−0.02	0.00	0.01	0.12	0.04	0.22
**4.5**	0.02	0	−0.01	−0.06	0.01	0.03	0.10	−0.06	0.12
**5.0**	−0.01	0	0.01	−0.07	0.00	0.04	0.08	−0.05	0.13
**5.5**	0.08	0	−0.04	0.09	0.00	−0.05	0.12	0.01	−0.01
**6.0**	−0.09	0	0.05	0.06	−0.01	−0.03	−0.03	0.12	0.03
**6.5**	0.08	0	−0.04	0.10	0.00	−0.06	0.11	0.02	−0.02
**7.0**	0.02	0	−0.01	−0.09	0.01	0.05	−0.18	−0.08	−0.16
**7.5**	0.13	0	−0.08	0.00	0.01	0.00	−0.05	−0.10	−0.19
**8.0**	0.11	0	−0.06	0.10	0.00	−0.06	−0.09	0.00	−0.27
**8.5**	0.04	0	−0.02	0.08	0.00	−0.05	−0.06	0.03	−0.15
**9.0**	0.04	0	−0.02	0.03	0.00	−0.02	−0.08	−0.01	−0.15
**9.5**	−0.02	0	0.01	0.07	−0.01	−0.04	−0.08	0.07	−0.11
**10.0**	0.06	0	−0.03	0.10	0.00	−0.06	−0.01	0.04	−0.13

Supplementary Table A2 | Element edge length and total number of elements of generated meshes of the mandible used for mesh convergence study. The chosen mesh is highlighted in grey.

Element edge length	Total number of elements	Refinement Ratio
8 mm	113481	
6 mm	153814	1.36
3.5 mm	216003	1.40
2.5 mm	271928	1.26
**1.8 mm**	**360489**	**1.33**
1.5 mm	437224	1.21

Supplementary Table A3 | Muscle forces used for baseline model based on (Nelson 1986) and (Korioth und Hannam 1990), weighted for unilateral clenching scenario with left being the working side and right the balancing side

		**Maximum muscle force [N]**	Fiber activation		Direction cosine				Resulting forces					
			left	right	right x	left x	y	z	right F_x	right F_y	right F_z	left F_x	left F_y	left F_z
Superficial Masseter	SM	**190.4**	0.7	0.6	−0.2	0.2	−0.4	0.9	−24	−48	101	28	−57	121
Deep Masseter	DM	**81.6**	0.7	0.6	−0.5	0.5	0.4	0.8	−27	18	37	32	21	45
Medial Pterygoid	MP	**174.8**	0.8	0.6	0.5	−0.5	−0.4	0.8	51	−39	83	−71	−55	116
Anterior Temporalis	AT	**158.0**	0.7	0.6	−0.1	0.1	0.0	1.0	−14	4	91	17	5	114
Medial Temporalis	MT	**95.6**	0.7	0.7	−0.2	0.2	0.5	0.8	−14	32	54	14	32	53
Posterior Temporalis	PT	**75.6**	0.6	0.4	−0.2	0.2	0.9	0.5	−6	25	14	9	38	21
Inferior lateral Pterygoid	ILP	**66.9**	0.3	0.7	0.6	−0.6	−0.8	−0.2	27	−33	−8	−13	−15	−3

2 Supplementary Figures

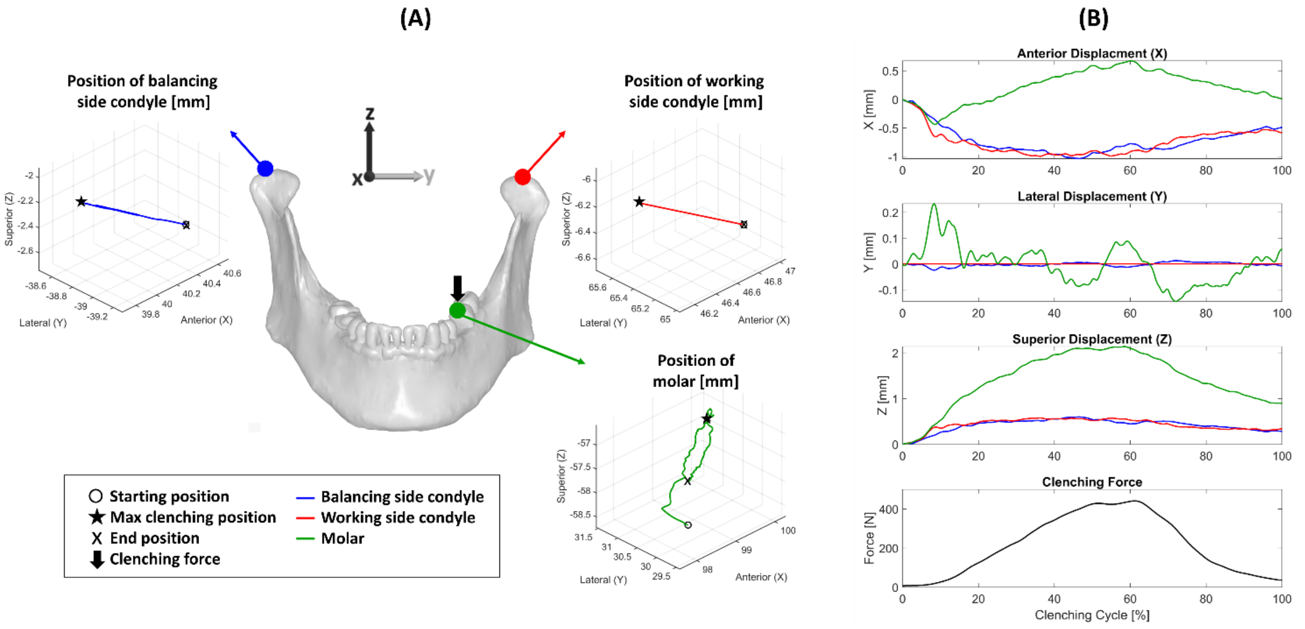

Supplementary Figure A1 | Three-dimensional position and displacements of the balancing and working side temporomandibular joints (blue and red) and working side left first molar (green) during the clenching cycle. **(A)** The 3D curves illustrate the spatial motion paths of each landmark, capturing the combined anterior (X), lateral (Y), and superior (Z) components throughout the cycle. Black circles denote the starting point of the cycle, black crosses the end point and the star the point where the maximum clenching force is applied. **(B)** Displacement trajectories are shown for each landmark normalized to the clenching cycle (%) and relative to their initial position. The bottom panel shows the corresponding clenching force profile.

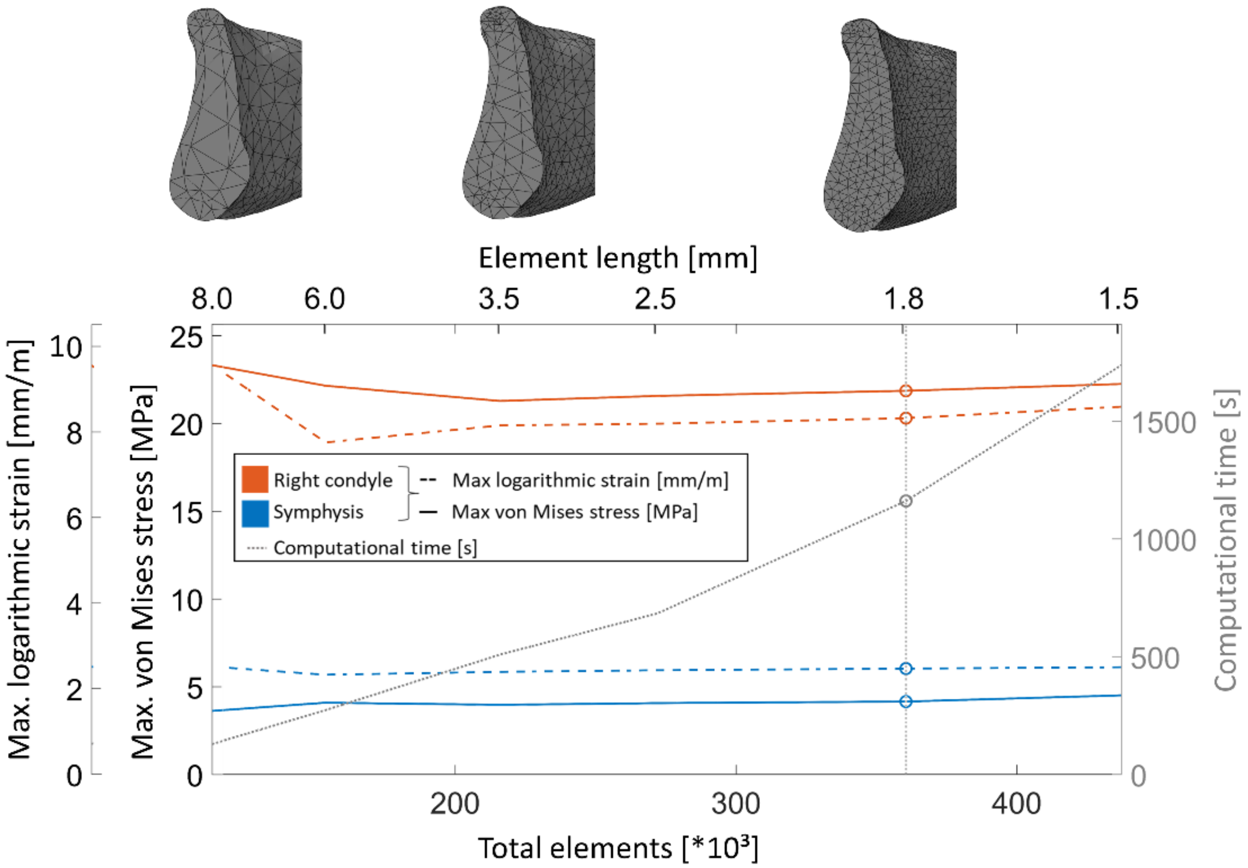

Supplementary Figure A2 | Mesh Convergence study on maximum von Mises stress (solid line) and strain (dotted line) in two regions of interest: Balancing side condyle (red) and symphysis (blue). The computational time is shown in grey. The horizontal line marks the final mesh size of 1.8 mm following the checklist from (Oefner et al. 2021)
